# In-Silico Evaluation of Genetic Alterations in Ovarian Carcinoma and Therapeutic Efficacy of NSC777201, as a Novel Multi-Target Agent for TTK, NEK2, and CDK1

**DOI:** 10.3390/ijms22115895

**Published:** 2021-05-31

**Authors:** Harshita Nivrutti Khedkar, Yu-Chi Wang, Vijesh Kumar Yadav, Prateeti Srivastava, Bashir Lawal, Ntlotlang Mokgautsi, Maryam Rachmawati Sumitra, Alexander T. H. Wu, Hsu-Shan Huang

**Affiliations:** 1 PhD Program for Cancer Molecular Biology and Drug Discovery, College of Medical Science and Technology, Taipei Medical University, Academia Sinica, Taipei 11031, Taiwan; harshitakhedkar@gmail.com (H.N.K.); bashirlawal12@gmail.com (B.L.); d621108006@tmu.edu.tw (N.M.); maryamrachma60@gmail.com (M.R.S.); 2 Graduate Institute for Cancer Biology & Drug Discovery, College of Medical Science and Technology, Taipei Medical University, Taipei 11031, Taiwan; 3 Department of Obstetrics and Gynecology, Tri-Service General Hospital, National Defense Medical Center, Taipei 11490, Taiwan; yuchitsgh@gmail.com; 4 The Program for Translational Medicine, Graduate Institute of Biomedical Informatics, College of Medical Science and Technology, Taipei Medical University, Taipei 11031, Taiwan; vijeshp2@gmail.com (V.K.Y.); prateetis2@gmail.com (P.S.); 5 Division of Gastroenterology and Hepatology, Department of Internal Medicine, Shuang Ho Hospital, New Taipei City 23561, Taiwan; 6 Division of Gastroenterology and Hepatology, Department of Internal Medicine, School of Medicine, College of Medicine, Taipei Medical University, Taipei 110, Taiwan; 7 The PhD Program for Translational Medicine, College of Medical Science and Technology, Taipei Medical University, Taipei 11031, Taiwan; 8 TMU Research Center of Cancer Translational Medicine, Taipei Medical University, Taipei 11031, Taiwan; 9 Clinical Research Center, Taipei Medical University Hospital, Taipei Medical University, Taipei 11031, Taiwan; 10 Graduate Institute of Medical Sciences, National Defense Medical Center, Taipei 11490, Taiwan; 11 National Defense Medical Center, School of Pharmacy, Taipei 11490, Taiwan; 12 PhD Program in Drug Discovery and Development Industry, College of Pharmacy, Taipei Medical University, Taipei 11031, Taiwan

**Keywords:** ovarian carcinoma, prognostic gene signature, bioinformatics, genetic alterations, drug resistance, protein-ligand interactions, target-based structure discovery

## Abstract

Ovarian cancer is often detected at the advanced stages at the time of initial diagnosis. Early-stage diagnosis is difficult due to its asymptomatic nature, where less than 30% of 5-year survival has been noticed. The underlying molecular events associated with the disease’s pathogenesis have yet to be fully elucidated. Thus, the identification of prognostic biomarkers as well as developing novel therapeutic agents for targeting these markers become relevant. Herein, we identified 264 differentially expressed genes (DEGs) common in four ovarian cancer datasets (GSE14407, GSE18520, GSE26712, GSE54388), respectively. We constructed a protein-protein interaction (PPI) interaction network with the overexpressed genes (72 genes) and performed gene enrichment analysis. In the PPI networks, three proteins; TTK Protein Kinase (TTK), NIMA Related Kinase 2 (NEK2), and cyclin-dependent kinase (CDK1) with higher node degrees were further evaluated as therapeutic targets for our novel multi-target small molecule NSC777201. We found that the upregulated DEGs were enriched in KEGG and gene ontologies associated with ovarian cancer progression, female gamete association, otic vesicle development, regulation of chromosome segregation, and therapeutic failure. In addition to the PPI network, ingenuity pathway analysis also implicate TTK, NEK2, and CDK1 in the elevated salvage pyrimidine and pyridoxal pathways in ovarian cancer. The TTK, NEK2, and CDK1 are over-expressed, demonstrating a high frequency of genetic alterations, and are associated with poor prognosis of ovarian cancer cohorts. Interestingly, NSC777201 demonstrated anti-proliferative and cytotoxic activities (GI_50_ = 1.6 µM~1.82 µM and TGI_50_ = 3.5 µM~3.63 µM) against the NCI panels of ovarian cancer cell lines and exhibited a robust interaction with stronger affinities for TTK, NEK2, and CDK1, than do the standard drug, paclitaxel. NSC777201 displayed desirable properties of a drug-like candidate and thus could be considered as a novel small molecule for treating ovarian carcinoma.

## 1. Introduction

Ovarian cancer ranks as the sixth most common cancer and most lethal of all gynecologic malignancies worldwide [1]. Since the ovary is deep in the pelvis, 70% of cases are diagnosed at an advanced stage with distant metastases. Within 2 years, most patients undergo disease recurrence and relapsed ovarian cancer lacks successful care choices. Therefore, ovarian cancer mortality rates rank first among gynecological malignant tumors [2]. Hence, it is of great importance to look for effective tumor markers and research their functions in ovarian cancer in order to explain the pathogenesis, diagnosis, prevention, and treatment. Long-term survival in ovarian cancer remains poor as a consequence of drug resistance, which results in fatal disease [3]. Intrinsic and acquired drug resistance occurs because of somatic cell genetic differences in tumors and individual variations in patients [4]. Genes, whose activation leads to cancer development known as oncogenes, also account for drug resistance in cancer [5]. For example, in chondrosarcomas, the leukemia/lymphoma-related factor, the LRF gene is an oncogene related to the survival factor and contributes to drug resistance and tumor malignancy [6].

In ovarian cancer, increased DNA damage tolerance/repair, enhanced antiapoptotic regulator activity, reduced cell-associated drugs, altered drug inactivation, and growth factor receptor deregulation account for drug resistance [7,8]. Drug resistance is caused by abnormal expressions of genes associated with drug resistance, which are key players among all of the oncogenes. On the basis of differentially expressed genes (DEGs) identified through the Gene Expression Omnibus (GEO) portal, it was found that elevated NEK2 gene expression was linked to drug resistance in ovarian cancer, compared to the parental counterparts [3]. It was reported that NEK2 (NIMA-related kinase 2) is responsible for tumorigenesis, progression, chromosome instability, and drug resistance in cancer [9,10]. Increased cyclin-dependent kinase (CDK) activity results in alterations of DNA damage and mitotic checkpoints, which drive the cell cycle [11]. Deregulated CDK activation causes unscheduled proliferation along with chromosomal and genomic instability [12]. CDK-cyclin complexes continue either proliferation or unplanned re-entry into the cell cycle, which is frequently observed with deregulation seen in certain CDK-cyclin complexes [13]. Specific CDKs are required by tumor cells for progression; hence, therapeutic strategies responsible for CDK inhibition should be taken into account depending on these particular criteria [14].

The dual-specificity protein kinase (TTK), a prognostic biomarker in liver cancer patients, is controlled by the mitotic spindle assembly control point and cell cycle program. Elevated expression levels of TTK in neoplastic tissues in a cohort of liver cancer patients were observed compared to neighboring healthy liver tissues. These heightened expression levels were associated with an aggressive clinical course and poor survival [15]. TTK, which phosphorylates serines/threonines and tyrosines, is also known as monopolar spindle 1 (Mps1). A key section of the spindle assembly checkpoint (Sac) is to ensure healthy cell proliferation and correct division of Mps1. In addition, it plays an important role in centrosome duplication and organ development [16]. Moreover in thyroid carcinoma, glioblastomas, and breast cancer, higher expression levels of TTK were seen [17,18,19,20].

As upregulation of these genes is associated with chromosome and genomic instability, finding a novel specific inhibitor that can target these genes become relevant. Emphasis has been laid on the identification and development of multi-target small molecules for cancer therapy [21]. Molecular docking analysis is a computational simulation of receptor-ligand interactions and has aided the process of target identification and drug discovery [22,23]. In line with the search for novel multitarget anti-cancer small molecules, we reported NSC777201, a novel small molecule for anticancer activity against ovarian cancer, and provided a structural insight of its multi-target potential against some identified oncogenic drivers of ovarian cancer [24,25,26]. NSC777201 demonstrated antiproliferative and dose-dependent anticancer activity against the NCI’s ovarian cancer cell lines, and its further evaluation towards TTK, NEK2, and CDK1 inhibition was carried out with in silico docking in a receptor-ligand interaction study. These lines of evidence provided the basis for NSC77201 directly regulating activities of TTK, NEK2, and CDK1, which result in antitumor effects in multiple cancer types. In this study, we mined the ovarian cancer gene signatures associated with chromosome instability (CIN) and genomic instability (GIN), female gamete formation, reproductive processes, and salvage pathways. Reversion of these gene signatures by our novel small molecule, NSC777201, may provide a target-based therapeutic effect towards ovarian cancer.

## 2. Results

### 2.1. Identification of a Common Gene Signature in Ovarian Cancer

**Figure 1:**
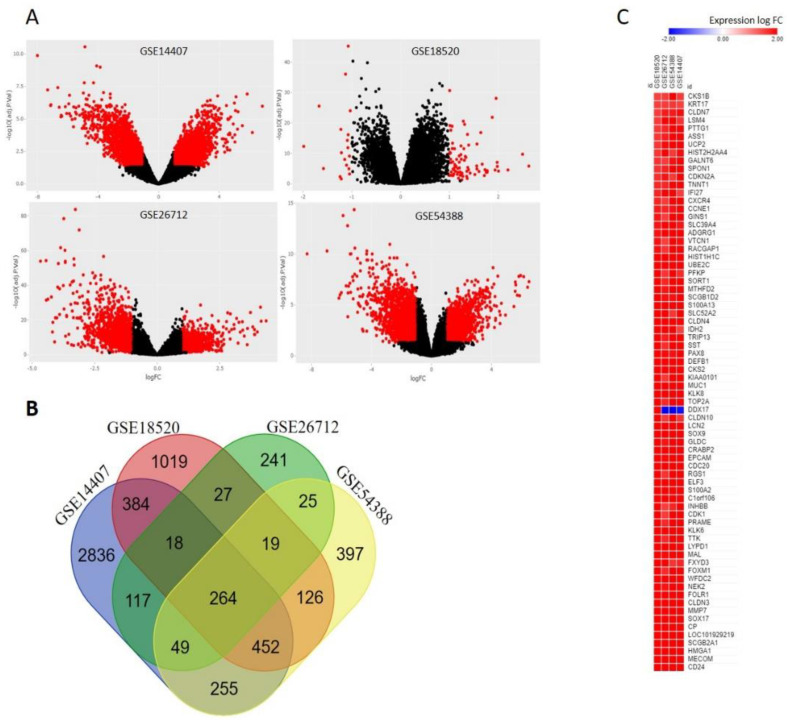
Identification of differentially expressed genes (DEGs). (**A**) Differential expression of genes between cancerous and non-cancerous samples in the datasets; GSE14407, GSE18520, GSE26712, GSE54388, the red data points in the volcano plot represent upregulated and downregulated genes screened based on |LOG FC| > 1.5 and a corrected *p*-value of <0.05. The black data points represent genes with no significant difference. The datasets were obtained from gene expression omnibus. (**B**) Venn diagram of common DEGs in the 4 datasets; a total of 264 DEGs overlapped in all 4 datasets. (**C**) The heat map of relative common upregulated gene signature and the GEO datasets. Each column in the figure represents a GEO dataset and each row represents a gene. The colors in the graph explain the magnitude of gene expression in datasets. The red color indicates that the gene is highly expressed in the dataset and the blue indicates that the gene expression is low.

Microarray datasets (Table 1) were retrieved from the NCBI-GEO database to identify the common intersected gene signature associated with ovarian cancer within all the datasets [27]. As described in Figure 1A, volcano plots showing the total numbers of DEGs obtained for all the datasets respectively. In GSE14407, 2191 DEGs including 1291 upregulated and 900 downregulated; in GSE54388, 1022 DEGs including 392 upregulated and 1020 downregulated; in GSE18520, 24 DEGs, including 21 upregulated and 3 downregulated; GSE26712, 381 DEGs including 105 upregulated and 276 downregulated were identified. Common intersected DEGs were obtained by using the web-based draw venn diagram platform. A total of 264 common DEGs were observed in ovarian carcinoma as compared to the normal adjacent tissues (Figure 1B) (Supplementary Material File S1). After a comprehensive analysis of the 4 datasets, 264 DEGs were found in all of them to be differentially expressed, among which 72 genes (Supplementary Material Table S1) were upregulated and 192 genes (Supplementary Material Table S2, Figure S1) were downregulated in ovarian carcinoma compared to those in normal ovary tissue. The common intersected upregulated genes (Figure 1C) were the main focus of the present study and were further analyzed in more detail as reported below. However, the diagnostic and prognostic role of the intersected downregulated genes in ovarian cancer is currently under investigation.

### 2.2. PPI Clustering Network Revealed Multiple Interactions of TTK, NEK2, and CDK1 with Oncogenic Proteins

**Figure 2:**
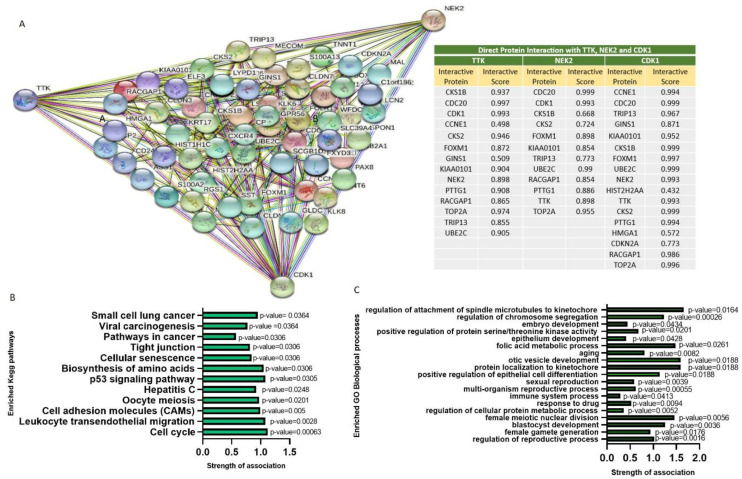
Protein-protein interaction (PPI) network visualization of TTK, NEK2, and CDK1. (**A**) Clustering network of TTK, NEK2, and CDK1 interactions generated 72 nodes and 166 edges with an average local clustering coefficient of 0.486 and PPI enrichment *p* < 10^−16^. Accompanying table shows the proteins interacting with TTK, NEK2, and CDK1 representing the highest-scoring interacting link to 0.999 (**B**) The Kegg pathways (**C**) Biological processes associated with TTK, NEK2, and CDK1 clustering networks.

Common upregulated DEGs from the four datasets were used to generate the PPI network to identify the highest possible number of interacting proteins. Clustering networks of TTK (TTK protein kinase), NEK2 (NIMA-related kinase 2), and CDK1 (cyclin-dependent kinase 1) generated 72 nodes and 166 edges with an average local clustering coefficient of 0.486 and PPI enrichment of *p* < 10^−16^ (Figure 2A). In Figure 2A accompanying table of network analysis, TTK directly interacted with 14 proteins with interactive scores of 0.498~0.997. The most profoundly interacting proteins with TTK were CDK1, CKS2, CDC20, and TOP2A. NEK2 had close interactions with 12 proteins, among which it directly interacted with UBC2C, TOP2A, CDK1, and CDC20 with combined scores of 0.854~0.999. CDK1 had 17 total interactions, and the most closely interacting proteins were TRIP13, NEK2, FOXM1, UBE2C, TTK, CKS2, TOP2A, CDC20, CKS1B, and CCNE1 with combined scores ranging 0.432~0.999. According to the KEGG, the strongly associated pathways with the TTK, NEK2, and CDK1 network involved the cell cycle, leukocyte transendothelial migration, biosynthesis of amino acids, p53 signaling pathway, cellular senescence, cell adhesion molecules (CAMs), viral carcinogenesis, hepatitis C, tight junctions, pathways in cancer, small cell lung cancer, and oocyte meiosis (Figure 2B) (Supplementary Material Table S3). The topmost related biological processes in the TTK, NEK2, and CDK1 network involved regulation of reproductive processes, female gamete generation, blastocyst development, female meiotic nuclear division, and regulation of cellular protein metabolic processes. Along with response to drugs, mitotic cell cycle processes, multi-organism reproductive processes, sexual reproduction, positive regulation of epithelial cell differentiation, protein localization to kinetochores, otic vesicle development, aging, folic acid metabolic processes, epithelium development, positive regulation of protein serine/threonine kinase activity, embryo development, immune system processes, and regulation of attachment of spindle microtubules to kinetochores were also included (Figure 2C) (Supplementary Material Table S4).

### 2.3. Canonical Pathway Analysis Maximized the Biological Interpretation in Terms of Disease and Functions

**Figure 3:**
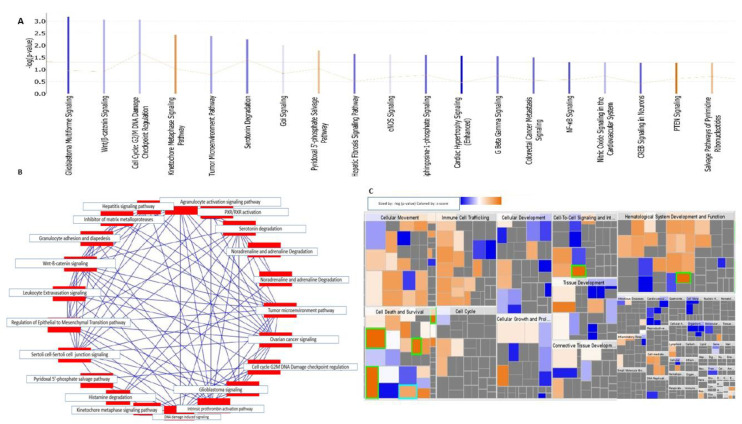
Maximizing the biological interpretation of genes with the -ngenuity pathway analysis (IPA): (**A**) The bar chart shows the names of the canonical pathways on the *x*-axis. The *y*-axis represents the negative log of the *p*-value of overlap. The orange bar indicates activated pathways, the blue bar indicates inhibited pathways, and the white bar indicates (**B**) Canonical pathway showing significant overlap with each pathway predicted (**C**) Each rectangle of the heat map represents a disease and functional category and most significantly enriched categories have largest rectangles.

The top canonical pathways enriched with common DEGs, were analyzed using the QIAGEN’s ingenuity pathway analysis tool, and are represented as a bar chart (Figure 3A). IPA mapped the significant canonical pathways affected by the DEGs, where the activation of Kinetochore metaphase, pyrimidine 5’-phosphate salvage, PTEN signaling, salvage pathways of pyrimidine ribonucleotides, and MSP-RON signaling in cancer cells were identified (Figure 3A). Diverse genetic alterations induced by endogenous as well as exogenous genotoxic agents such as free radicals, chemicals, by-products of intracellular metabolism, ionizing radiation, or medical therapy [28]. The DNA damage checkpoints play an important role in protecting cells from these constant attacks. Cells can go through senescence or apoptosis if the repair is unsuccessful in DNA repair machinery or at the checkpoint. Ultimately cells will accumulate the DNA alterations leading to genomic instability and causing the cell transformation and ontogenesis [28]. Spindle microtubules are attached to chromosomes by kinetochore, which confirms the attachment and stability of microtubule status to the spindle assembly checkpoint, a signaling pathway responsible to correct chromosome segregation and modulation of CDK1 activity [29,30]. The canonical pathway findings suggest that the upregulation in the kinetochore-signaling pathway due to the differentially expressed genes leads to genomic and chromosomal instability, which in turn contributes to the tumor progression. The taller the bar, the more significant the *p*-value. In IPA, the bars are arranged in order of significance so the most significant pathways are located at the leftmost side of the bar chart. (Figure 3A). The shared biology among candidate genes identified is represented in the overlapping canonical pathways map. Many or one genes in particular share the connected canonical pathways. The brighter the red color of the node, the significant is the canonical pathway in the collection of genes. The canonical pathways map created using the ingenuity pathway analysis tool from QIAGEN (Figure 3B). In the IPA system, the data can be visualized as the integration of gene expression and small-scale experiments [31,32]. The network of interaction between molecules, including genes, proteins chemicals, and drugs can be visualized in the experimental system. IPA uncovers the interaction and functional roles of the DEGs associated with the signaling pathways and their related disease and functions [33]. The disease and function classification given in Figure 3C indicated that the DEGs are associated with the activation of cellular movement, immune cell trafficking, cell death and survival, cell cycle, cellular development, cellular growth and proliferation, cell to cell signaling, tissue development, connective tissue development, and hematological system development and function.

### 2.4. Overexpression of TTK, NEK2, and CDK1 Predicts a Poor Prognosis in Ovarian Cancer Patients

**Figure 4:**
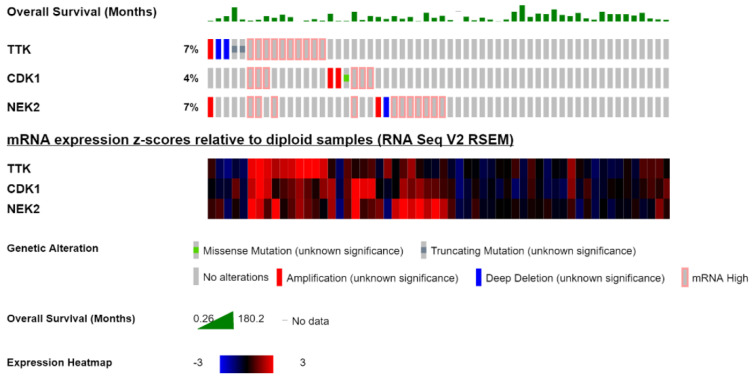
Genetic alteration analysis and OS towards TTK, NEK2, and CDK1 genes through a cBioportal analysis. TTK, NEK2, and CDK1 expressions in ovarian cancer. A bar code plot (OncoPrint) for genetic alterations, mutation status, copy number alterations, mRNA expressions, and OS of TTK, NEK2, and CDK1 genes in ovarian cancer were analyzed using the cBioPortal cancer genomics database.

To investigate the roles of TTK, NEK2, and CDK1 overexpression, we mined the TCGA’s ovarian cancer genomics data via the cBioportal website [34,35]. Patients with complete genomics data harbored amplification, deep deletion, and truncating mutations, which tended to have higher mRNA levels of TTK, NEK2, and CDK1 expressions. Dual specificity protein kinases, TTK were involved in the control of the cell cycle program, TTK is considered an important biomarker in liver cancer, triple-negative breast cancer, ovarian cancer, gastric cancer, and colorectal cancer [15,36,37,38,39]. High expression of TTK leads to aneuploidy and is concerned with aggressive subgroups and poor survival. In triple-negative breast cancer and other aggressive cancers of the breast subgroups, the TTK protein is a prognostic marker, and treatment resistance and aggressiveness of the cancer are due to the protection of the CIN caused by proteins like TTK. Hence, targeting overexpression of TTK could be therapeutic as well as could lead to significant survival [38]. Overexpression of TTK in hepatocellular carcinoma corresponded with hepatitis B surface antigen (HBsAg), age, satellite lesions, and the Edmondson tumor grade [40]. As described in the previous study of ovarian cancer patients, the disease-free survival (DFS) rate and overall survival rate were used to analyze the prognostic value of TTK (Figure 4). In addition, a Kaplan-Meier analysis model showed that overexpression of TTK was significantly associated with poor DFS (*p* = 0.033) [37] and OS (*p* = 0.018) [37]; these results indicate that TTK could be a potential biomarker.

Centrosome duplication and cell cycle regulation involve phosphorylation of proteins and constitute catalytic activity mediated by NEK2 [9]. During the G_2_/M phase of the cell cycle, NEK2 binds to microtubules, where it is responsible for centrosome splitting [9]. Premature splitting of organelles was caused by upregulation of NEK2 [41]. Centrosome abnormalities and aneuploidy were caused by overexpression of NEK2 kinase-dead mutants [42]. To ensure the cell cycle’s timely progression and correct centrosome duplication, strict operational regulation of NEK2 abundance is essential. In patients with pancreatic ductal adenocarcinoma, high expression levels of NEK2 were substantially correlated with lymph node metastasis (*p* = 0.003) and tumor stage (*p* = 0.001). Increased expression of NEK2 through univariate and multivariate analyses revealed NEK2 as an independent indicator of a poor prognosis in hepatocellular carcinoma [43]. Patients with high NEK2 expression had significantly worse OS relative to those with low NEK2 expression (Figure 4). This led to the identification of NEK2 as a promising prognostic biomarker [44]. NEK2 was linked to inferior survival and poor prognoses in different cancers such as T-cell acute lymphoblastic leukemia, head and neck squamous cell carcinoma, bladder carcinoma, glioblastomas, hepatocellular carcinoma, and ovarian adenocarcinomas [45]. Drug resistance in ovarian cancer is associated with the upregulation of NEK2 [3], along with that it is an oncogene whose activation leads to the development of cancer hence targeting its activity becomes crucial.

Upregulation of CDK1 was observed in various cancers like human colorectal cancer [46], gastric lymphoma [47], Hodgkin’s lymphoma [48], prostate cancer [49], childhood acute lymphoblastic leukemia [50], and ovarian cancer [51]; hence, CKD1 is associated with the prognosis of multiple malignant tumors. When organogenesis takes place in the early development of an embryo, where the cell division is the most active process, CDK1 alone is sufficient in most cellular lineages to drive cell division [52]. Unscheduled proliferation is caused by tumor cells accumulating mutations, which results in defective anti-mitogenic signals [53,54]. Numerical changes in chromosomes occur due to genomic instability (GIN) and chromosomal instability (CIN) acquired by most tumors due to mutations, a defect responsible for unscheduled proliferation [55]. Tumor progression of violent phenotypes and their acquisition is a result of increased susceptibility to the accumulation of genetic mutations. Deregulation of CDKs leads to GIN, CIN, and unscheduled proliferation defects in the cell cycle. Overexpression of CDK1 was shown to be associated with a worse prognosis in terms of 5-year OS (Figure 4) [56]. This indicates that suppression of CDK1 can reduce ovarian cancer growth.

### 2.5. Frequent Overexpression of the TTK, NEK2, and CDK1 Genes in Ovarian Cancer

**Figure 5:**
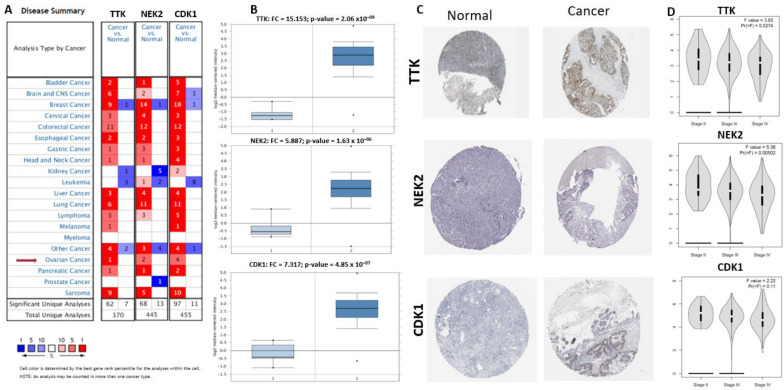
Expression profiles and roles of the TTK, NEK2, and CDK1 genes in ovarian cancer. (**A**) Expressions of TTK, NEK2, and CDK1 in all tumor studies in the Oncomine database. (**B**) Expressions of TTK, NEK2, and CDK1 in chips of different ovarian cancer studies within the Oncomine database. (**C**) Expressions of TTK, NEK2, and CDK1 in ovarian cancer tissues and normal tissues in the Human Protein Atlas. (**D**) Correlations between TTK, NEK2, and CDK1 expressions and tumor stage in ovarian cancer patients. From the GEPIA database, a violin plot was derived from correlations of TTK, NEK2, and CDK1 expressions with the tumor stage in patients with ovarian carcinoma; the *p*-value was set to 0.05. The abscissa indicates the stage of ovarian cancer, and the ordinate indicates expression levels of TTK, NEK2, and CDK1.

The Oncomine database was used for the expression analysis of TTK, NEK2, and CDK1 in ovarian cancer and normal samples [57]. The findings showed that in different cancers, CDK1 was overexpressed, and its expression was greatly increased in ovarian cancer patients. From 370, 445, and 455 different tumor studies, TTK, NEK2, and CDK1 gene expression data were collected. Of these, 62, 68, and 97 studies showed increased expressions of TTK, NEK2, and CDK1 (Figure 5A). Further, *TTK*, *NEK2*, and *CDK1* gene expressions were filtered from the TCGA ovarian statistics and were found to be higher than those in the normal group (Figure 5B). The HPA database was used to verify histological levels of TTK, NEK2, and CDK1, and results suggested that TTK, NEK2, and CDK1 were upregulated in ovarian cancer tissue compared to the normal tissue (Figure 5C) [34,58]. Furthermore, we used the GEPIA web-based tool [59] to investigate the roles of these genes in ovarian cancer; interestingly, we found that expressions of TTK, NEK2, and CDK1 were correlated with tumor stages. mRNA levels of TTK, NEK2, and CDK1 increased during the development of cancer from stage 1 to stage 4 (Figure 5D). This indicates that they have important functions in ovarian cancer tumorigenesis 2.06 × 10^–9^.

### 2.6. NSC777201 Met the Required Criteria of Drug Likeness, and Showed Antiproliferative and Dose-Dependent Cytotoxic Effects

Having identified the oncogenic role of TTK, NEK2, and CDK1 in ovarian cancer, and to contribute to the ongoing program in the development of anti-cancer drugs against ovarian cancer, we synthesize NSC777201 a derivative of our previously developed anticancer small molecule [26], evaluated its drug-likeness and its efficacy against both primary and multidrug resistance ovarian cancer cell lines. In addition, we provided a structural insight for targeting TTK, NEK2, and CDK1.

**Figure 6:**
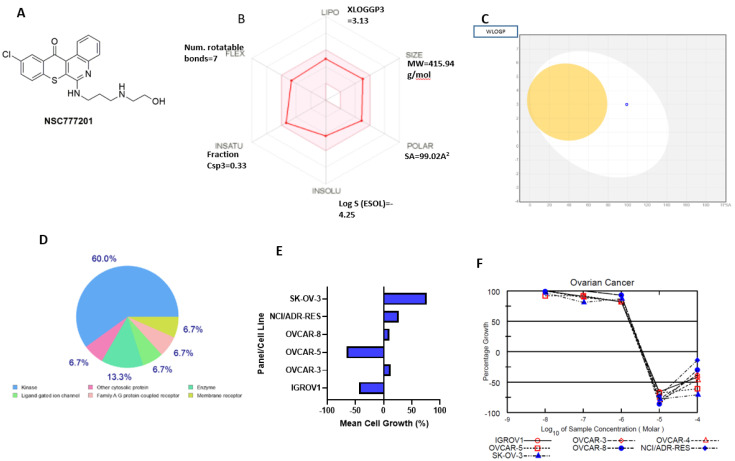
NSC777201 met the required criteria of drug-likeness. (**A**) Schematic representation of the structure of NSC777201. (**B**) Bioavailability radar presenting suitable physicochemical spaces of the oral bioavailability of NSC777201. The optimal range of each property is presented in the pink area. (**C**) BOILED-Egg model of the brain or intestinal estimated permeation of NSC777201. (**D**) Pie chart presents the repartition of protein classes of potential druggable candidates for NSC777201. (**E**) Antiproliferative effects of NSC777201 against the panel of NCI ovarian cancer cell lines. A single dose of 10 µM of NSC777201 was used to treat each cell line. The zero point denotes the mean percentage of cell growth. Each cell line’s percentage growth inhibition relative to the mean is represented by values under 100, and values below zero indicate cell death. (**F**) Dose-dependent cytotoxic response curves of NSC777201 against the panel of NCI’s ovarian cancer cell lines. The growth of untreated cells as the growth percentage value of +100 is presented on the *Y*-axis.

Consequently, we found that NSC777201 fulfilled the required criteria of a good drug candidate in terms of lipophilicity, polarity, flexibility, solubility, saturation, and molecular weight. The compound has demonstrated good synthetic accessibilities, highly probable GIA absorption, and bioavailability, but poor blood-brain barrier permeation (Figure 6A–C). The physicochemical properties, water-solubility, lipophilicity, pharmacokinetics, drug-likeness, and medicinal chemical properties of NSC777201 are presented in supplementary file S8. According to the OECD classification, the predicted environmental toxicity and acute toxicity for different administration routes of NSC777201 produce class 4 and 5 levels of acute toxicity (LD50) (Supplementary Material Table S5). Collectively, NSC777201 meets the criteria of drug-likeness and is relatively toxic. Drug target prediction software [60] showed that has a number of targetable proteins. Most of which were kinases and other classes of NSC777201 targetable proteins include ligand-gated ion channel, enzyme, and membrane receptors (Figure 6D).

NSC777201 exhibited anti-proliferative effects against the panel of NCI’s ovarian cancer cell lines (Figure 6E). In a multiple-dose screening, NSC777201 demonstrated anti-cancer activity with GI_50_ values of less than 2 µM. Interestingly, we found that both parental cell lines including OVCAR-5 (TGI = 3.5 µM) and IGROV1 (TGI = 3.6 µM), and the chemo-resistant cell lines including SK-OV-3 (TGI = 3.38µM), NCI/ADR-RES (TGI = 3.63 µM), OVCAR-8 (TGI = 3.32 µM), OVCAR-4 (TGI = 3.23 µM), and OVCAR-3 (TGI = 3.51 µM) demonstrates anti-proliferative response to NSC777201 treatments (Figure 6F) (Table 2). The SK-OV-3 and NCI/ADR-RES cell lines have been established to be resistant to chemotherapy including cyclophosphamide, Adriamycin, and cisplatin therapies [61,62].

### 2.7. In Silico Molecular Docking Indicates the Ligand-Receptor Interactions of NSC777201 with TTK, NEK2, and CDK1

**Figure 7:**
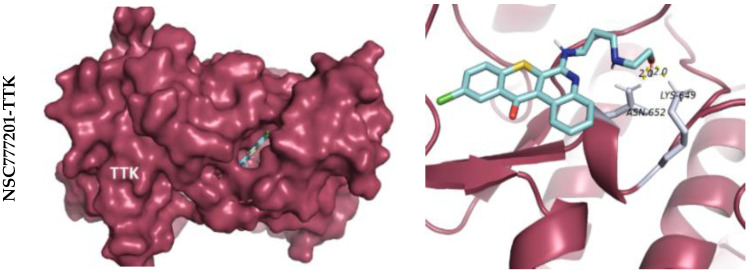

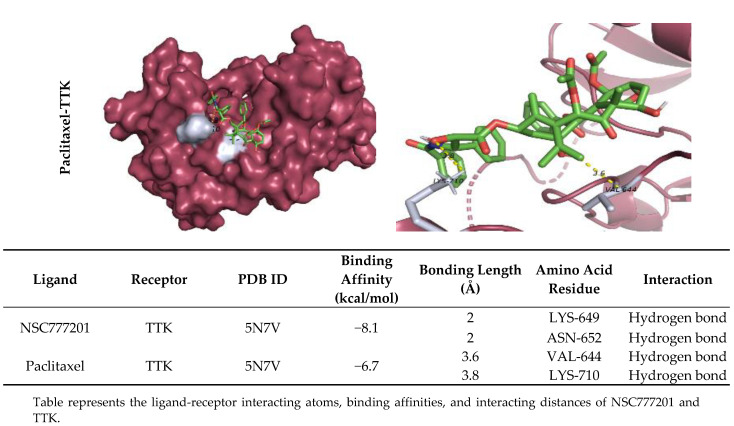
Docking profile of TTK with NSC777201 and paclitaxel (a known inhibitor). 3D structure of ligand-receptor interactions shown in the left panel. The right panel shows the 2D representation of the interaction with ligands and the receptors in the binding pocket.

**Figure 8:**
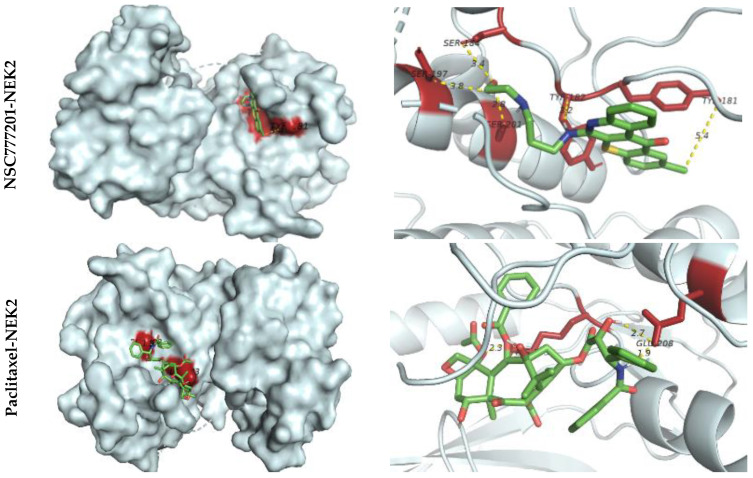

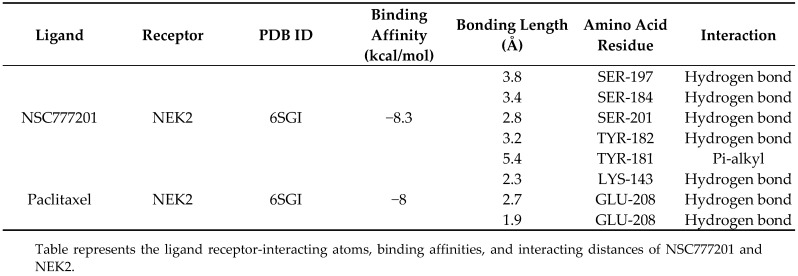
Docking profile of NEK2 with NSC777201 and paclitaxel (a known inhibitor). 3D structure of ligand-receptor interactions shown in the left panel. The right panel shows the 2D representation of the interaction with ligands and the receptors in the binding pocket.

**Figure 9:**
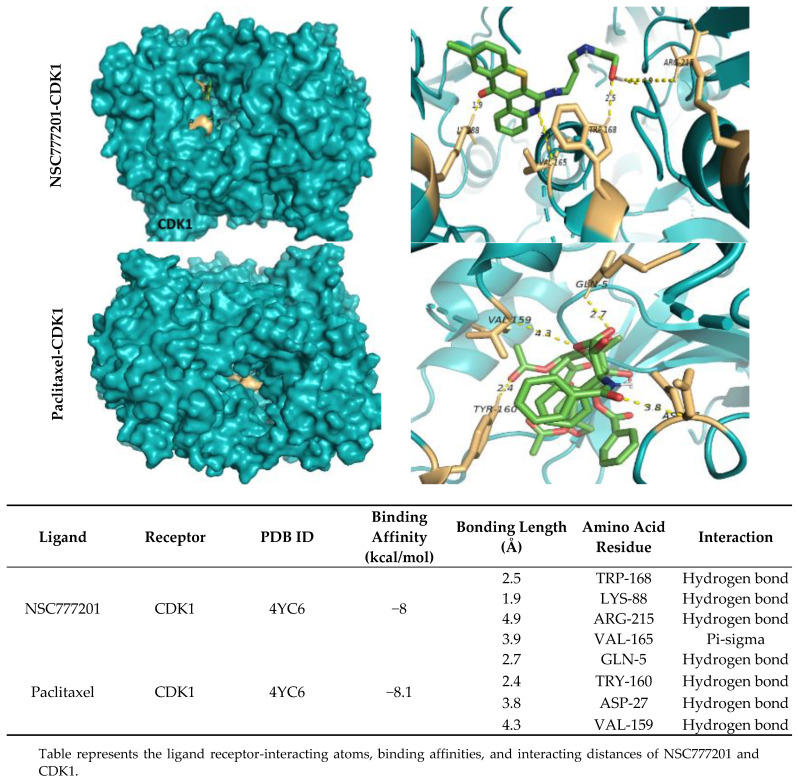
Docking profile of CDK1 with NSC777201 and paclitaxel (a known inhibitor). 3D structure of ligand-receptor interactions shown in the left panel. The right panel shows the 2D representation of the interaction with ligands and the receptors in the binding pocket.

A bioinformatics study followed by docking simulations determined possible interactions of NSC777201 with NEK2, TTK, and CDK1 (Figure 7, Figure 8 and Figure 9). The interaction analysis of NSC777201 and receptors in the binding pocket, exposed to NSC777201 interacted with NEK2 by four conventional H-bonds in close proximity of 3.8 Å, 3.4 Å, 2.8 Å, and 3.2 Å with SER197, SER184, SER201, and TYR182 residues, respectively, and was further stabilized by a pi-alkyl interaction in the proximity of 5.4 Å with TYR181. Results generated revealed that NSC777201 interacts with NEK2 with the lowest binding energy of −8.3 kcal/mol (Figure 7). Similarly, and TTK interacts with NSC777201 through 2 conventional H bonds with LYS649 and ASN652 in close proximity of 2.0 Å and 2.0 Å, respectively, with the binding energy of −8.1 kcal/mol (Figure 8). The CDK1-NSC777201 complex is stabilized by 3 H-bonds with TRP168, LYS88, and ARG215 in close proximity of 2.5 Å, 1.9 Å, and 4.9 Å, respectively, and 1 pi-sigma bond with VAL165 at a proximity of 3.9 Å with a binding energy of −8.0 kcal/mol (Figure 9). These binding energies compared to a known inhibitor, paclitaxel, were less negative in the case of NSC777201, and hence NSC777201 should be a potent inhibitor of NEK2, TTK, and CDK1.

## 3. Discussion

Ovarian cancer possesses a high metastatic capacity and high mortality. Relapse and resistance to current chemotherapeutic agents along with an alarming diagnosis at an early stage are the most important factors responsible for the high mortality rates in ovarian cancer according to data from The Surveillance, Epidemiology and End Results (SEER) Program of the National Cancer Institute [63]. Elucidation of the pathogenesis of ovarian cancer was pursued by identifying DEGs in tumor vs. normal samples from publically available databases. This provided promising biomarkers for targeted therapies and early diagnoses. To explore changes in a disease’s genetic aspects, high-throughput sequencing and microarray technologies are used in the field of genomics. As identified in the present study, TTK, NEK2, and CDK1 play significant roles in drug resistance, poor OS, and tumorigenesis. Mutations in kinase-coding genes are initiated through oncogenic transformations. The cellular aberration, tumorigenesis, is caused by mutations in these kinases. Thus, determining inhibitors of these upregulated kinases for effective clinical outcomes and cancer management is essential [64]. When subjected to a GO and KEGG pathway enrichment analysis, upregulated gene signatures in ovarian cancer indicated that they are mainly involved in female gamete formation, blastocyst development, reproductive processes, female meiotic nuclear division, regulation of chromosome segregation, organelle organization, and otic vesicle development in terms of biological processes in GO. Pathways affected by the upregulation of TTK, NEK2, and CDK1 involved the biosynthesis of amino acids, cellular senescence, cell adhesion molecules, viral carcinogenesis, and leukocyte transendothelial migration.

Regulation of biological processes involved in the attachment of spindle microtubules to kinetochores, regulation of cellular protein metabolic processes, immune responses, multi-organism reproductive processes, and sexual reproduction is affected by increased activities of TTK, NEK2, and CDK1. Through specific hormones, immature oocytes become fertilizable eggs by a process known as meiotic maturation. Activation of various signal transduction pathways that unite to activate the maturation-promoting factor (MPF) is needed for oocyte maturation, and this is a key movement for entering the M-phase of meiosis I and meiosis II [65]. An extensive network of feedback signaling is an important feature of meiotic maturation responsible for ensuring that an oocyte completes meiotic progression. Upregulation of TTK, NEK2, and CDK1 was associated with growth factor-initiated signaling, i.e., salvage pyrimidine and purine pathways are frequently deregulated in cancer. To assemble macromolecules like lipids, proteins, and nucleic acids for cell growth, higher anabolic metabolism is encouraged by these oncogenic modifications. Hence, these signaling pathways impact nucleotide metabolism, and the direct roles of TTK, NEK2, and CDK1 in regulating salvage pathways are recognized to be prominent in various forms of tumors [66]. Hence, the identification of novel small molecules, which can regulate the overexpression of these genes, becomes crucial. We have earlier reported NSC777201 for some biological activities [25,26,67], herein, we used a molecular docking approach and an NCI’s ovarian cancer cell lines to evaluate its anti-cancer activity and explore its possibility for targeting TTK, NEK2, and CDK1. Interestingly, we found that NSC777201 exhibited anti-proliferative and dose-dependent cytotoxic activity against the NCI’s ovarian cancer cell lines. Hence could be considered a novel small molecule with a potential reputation in the treatment of ovarian cancer.

On the basis of predictions made with in silico Swiss Target, it was suggested that kinases were identified as the most probable targets for NSC777201, which was further confirmed by examining ligand-receptor interactions. The close proximity and high binding affinities of NSC777201 to receptors suggest that NSC777201 is a better ligand for TTK, NEK2, and CDK1 than is paclitaxel, a known inhibitor. This significant binding of NSC777201 to receptors may be attributed to the larger number of H-bonds, and pi-sigma and pi-alkyl interactions. The concept of drug-likeness can play an important role in identifying potential drug candidates during early cancer stages [68]. In drug design and risk assessment of chemicals, it is important to estimate rodent acute toxicity (LD_50_), an adverse effect that follows a single-dose exposure [69]. Collectively, our study suggested that TTK, NEK2, and CDK1 are novel biomarker signatures of ovarian carcinoma and an attractive target for NSC777201 with consequent anticancer implications. NSC777201, therefore, serves as a novel small molecule worthy of further preclinical evaluation of its full therapeutic potential.

## 4. Materials and Methods

### 4.1. Data Collection and Preprocessing to Identify DEGs

The GEO database at the National Center for Biotechnology Information (NCBI; https://www.ncbi.nlm.nih.gov/geo/ (accessed on 16 October 2020) [27] contains normal and ovarian cancer tissue sample microarray datasets, viz., GSE14407, GSE18520, GSE26712, and GSE54388, was obtained for analysis. A volcano plot was generated with the limma package in R (vers. 3.2.5; https://www.r-project.org/ (Accessed on 6 November 2020), and the fold change (logFC) was calculated to screen DEGs between normal and cancerous ovarian tissues, with |LogFC| > 1.5 and the corrected *p*-value (*p* < 0.05) set as the cutoff criteria. A Venn diagram was generated using Draw Venn Diagram (http://bioinformatics.psb.ugent.be/webtools/Venn/ (Accessed on 11 November 2020)). A heatmap of upregulated genes was generated using Morpheus, online web software (https://software.broadinstitute.org/morpheus (Accessed on 14 December 2020)).

### 4.2. Clustering of Protein-Protein Interaction (PPI) Networks, Gene Ontology (GO), and Kyoto eEncyclopedia, Genes, and Genomes (KEGG) Pathway Analysis

Upregulated genes common in all four datasets were used to construct a PPI network by retrieving interacting genes through an online search using the tool STRING, vers. 11.0 (https://string-db.org/ (Accessed on 30 November 2021)) database. Functional protein partners in the PPI network, which regulated biological processes, were further identified.

### 4.3. Ingenuity Pathway Analysis (IPA)

The IPA system is a QIAGEN’s bioinformatics pathway analytical tool (https://www.qiagenbioinformatics.com/products/ingenuity-pathway-analysis (Accessed on 30 November 2021)) that uses a network generation algorithm to segment the network map of molecules into multiple networks and assign scores to each network [33]. For the canonical pathway, disease, and function analyses, a threshold of the Z-score of ≥2 was defined as significant activation, and a Z-score of ≤−2 was defined as significant inhibition [33]. Algorithms to calculate the overlap of Z-scores and *p* values were described previously [31].

### 4.4. Cancer Genome Exploration through the Computational Biology Center (cBio)

The prognostic (overall survival, OS) and genetic alteration (mutation, copy number variation, and messenger mRNA expression) data for ovarian cancer patients (“TCGA, PanCancer Atlas” dataset) were obtained from the cBioPortal (https://www.cbioportal.org/ (Accessed on 26 January 2021)) [34,35]. It is a tool for exploring, analyzing, and visualizing multidimensional cancer genomics data. Through the portal, we obtained readily understandable epigenetic, genetic, gene expression, and proteomic events.

### 4.5. TTK, NEK2, and CDK1 Expression Analysis through Various Databases

The world’s largest database of oncogene chips and integrated data-mining platform for cancer gene knowledge mining is currently the Oncomine database (https://www.oncomine.org (Accessed on 25 January 2021)), with 86,733 pieces of cancer tissues and normal tissues and 715 gene expression datasets having been collected [70,71]. The classification of differential expressions of cancer types and their respective normal tissues were analyzed on the basis of the Oncomine database. Large amounts of transcriptomics and proteomics data in specific human tissues are provided by the Human Protein Atlas (HPA) (https://www.proteinatlas.org/ (Accessed on 24 January 2021)) which is composed of the Cell, Pathology, and Tissue Atlas. The database offers information on 44 different normal tissue and organ cell-specific localization along with 20 of the most common types of cancer [71,72]. Using data from the HPA, immunohistochemical (IHC) expression maps of protein expression patterns in normal human tissues and tumor tissues were generated. mRNA expression levels of the TTK, NEK2, and CDK1 genes in ovarian cancer patients in the dataset of The Cancer Genome Atlas (TCGA) were analyzed using a gene expression profiling interactive analysis (GEPIA: https://gepia.cancer-pku.cn/ (Accessed on 24 January 2021)) [59].

### 4.6. Pharmacokinetics, Drug Likeness, Toxicity, and Medicinal Chemical Analyses of NSC777201

The pharmacokinetics, ADMET (adsorption, distribution, metabolism, excretion, and toxicity), and drug-likeness properties of NSC777201 were analyzed using the SwissADME algorithm [60]. Drug likeness was analyzed following the Lipinski (Pfizer) rule of five [73], while the Abbot Bioavailability score was used to estimate the drug’s oral bioavailability [74]. The Brain or Intestinal EstimateD permeation (BOILED-Egg) model was employed to predict blood-brain barrier penetration and gastrointestinal absorption (GIA) properties [75]. The environmental toxicity and acute toxicity in rats were predicted using GUSAR software [76].

### 4.7. In Vitro Dose-Dependent, Anticancer Screening Analysis of NSC777201

The anticancer properties of NSC777201 were evaluated against a panel of NCI’s ovarian cancer cell lines comprised of SK-OV3, NCI/ADR-RES, OVCAR-8, OVCAR-5, OVCAR-3, and IGROV1. As per NCI protocols [77,78], 10,000~20,000 cells/well were seeded in 96-well plates for 24 h followed by single-dose treatment with NSC777201 at 10 µM and incubation at 37 °C in 5% humidified CO_2_ for 48 h. A sulforhodamine B (SRB) [79] solution was used to fix cells, followed by a series of washing and staining to determine their viability. Growth inhibition was calculated relative to cells without drug treatment and the time-zero control. Following a single-dose screening, NSC777201 was further evaluated for dose-dependent activities at concentrations of 0.01, 0.1, 1.0, 10, and 100 µM. Results are presented in terms of total growth inhibition (TGI), the concentration needed to kill 50% of cancer cells (LC_50_), and the concentration needed to inhibit 50% of cancer cell growth (GI_50_) [80].

### 4.8. In Silico Molecular Docking Analysis

The three-dimensional (3D) structure of NSC777201 was drawn in sybyl mol2 using the Avogadro molecular builder and visualization tool version 1.1.0 [81]. Using the PyMOL Molecular Graphics System, vers. 1.2r3pre (Schrödinger, LLC, Palo Alto, CA2002, USA), the structure was transformed into the protein databank (PDB). The 3D structure of the receptors and crystal structures of TTK (PDB; 5N7V), NEK2 (PDB; 6SGI), and CDK1 (PDB; 4YC6) were retrieved from the PDB. The PDB file formats of the ligands (NSC777201 and paclitaxel) and receptors (TTK, NEK2, and CDK1) were converted to Auto Dock Pdbqt format using AutoDock Vina (vers. 0.8, The Scripps Research Institute, La Jolla, CA, USA) [82]. The removal of water molecules, the addition of hydrogen atoms, and Kolmman charges in the receptor were made as prerequisites of pre-docking. Molecular docking studies were conducted using AutoDock VINA software and by following protocols described in our previous studies [67,83,84,85]. The best poses of ligand-receptor complexes of hydrogen bonds and electrostatic and hydrophobic interactions were expressed as binding energy values (kcal/mol) to represent docking results. To visualize H-bond interactions, binding affinities, interacting amino acid residues, atoms binding to the ligands and receptors, and 3D graphical representations of ligand-receptor complexes were made using PyMOL software.

### 4.9. Data Analysis

Pearson’s correlations were used to assess correlations of differentially expressed genes. The statistical significance of DEGs was evaluated using the Wilcoxon test. * *p* < 0.05 was accepted as being statistically significant. The growth inhibition by NSC777201 in a single dose assay was obtained by subtracting the positive value on the plot from 100, i.e., a value of 60 would indicate 40% growth inhibition. Genetic alterations were calculated based on the c-bioportal web tool instructions. The adjusted value < 0.05 was considered statistically significant. All *p*-values were denoted as * *p* < 0.05; ** *p* < 0.01; *** *p* < 0.001.

## 5. Conclusions

In conclusion, TTK, NEK2, and CDK1 are strongly associated with tumorigenesis, therapeutic resistance, and poor prognosis of ovarian carcinoma and thus serve as a novel biomarker for diagnosis as well as attractive therapeutic targets for the treatment of ovarian carcinoma. Our study has contributed to the understanding of the development and pathogenesis of ovarian cancer. In addition, we reported a novel small molecule, NSC777201 with anti-proliferative and dose-dependent activity against NCI’s ovarian cancer cell lines. In addition, we provided structural base evidence indicating NSC777201 as a multi-target for TTK, NEK2, and CDK1.

## Figures and Tables

**Table 1:** Microarray datasets of ovarian cancer patients.

Accession No.	Platform	No. of Cases	
		Normal	Tumor
GSE18520	HG-U133_Plus_2 ^1^	10	53
GSE26712	HG-U133A ^2^	10	185
GSE54388	HG-U133_Plus_2 ^1^	6	16
GSE14407	HG-U133_Plus_2 ^1^	12	12

^1^ Affymetrix Human Genome U133 Plus 2.0 Array (GPL570). ^2^ Affymetrix Human Genome U133A Array (GPL96).

**Table 2:** Cytotoxic and anti-proliferative activities of NSC777201 against NCI’s ovarian cancer cell lines.

Cell Lines	GI_50_ µM	TGI µM	LC_50_ µM
IGROV1	1.6	3.6	>100
OVCAR-3	1.79	3.51	>100
OVCAR-4	1.6	3.23	>100
OVCAR-5	1.63	3.5	7.53
OVCAR-8	1.75	3.32	>100
NCI/ADR-RES	1.82	3.63	>100
SK-OV-3	1.68	3.38	6.8

G1_50_ = concentration needed to inhibit 50% of cancer cell growth, TGI = total growth inhibition (TGI), LC_50_ = concentration needed to kill 50% of cancer cell.

## Data Availability

The datasets generated and/or analyzed in this study are available on reasonable request.

## Supplementary Materials

The following are available online at https://www.mdpi.com/article/10.3390/ijms22115895/s1, File S1: Differentially expressed genes (DEGs) prepared from microarray datasets.
