# Supplementary material for: In-Silico Evaluation of Genetic Alterations in Ovarian Carcinoma and Therapeutic Efficacy of NSC777201, as a Novel Multi-Target Agent for TTK, NEK2, and CDK1
Source: Int J Mol Sci. 2021 May 31;22(11):5895. doi: 10.3390/ijms22115895 (PMC8198179; doi:10.3390/ijms22115895)
Supplement: Supplementary file 1 [file ijms-22-05895-s001.zip › suppl/ijms-1180317 suppl.pdf]

Table S1.	The common upregulated gene signature in ovarian cancer and gene FC values.				
	GSE18520	GSE26712	GSE54388	GSE14407	Average FC
**CKS1B**	1.5035113	1.5564532	2.41202543	1.54846723	1.75511429
**KRT17**	1.5232635	1.5638609	1.57988719	1.50876206	1.543943413
**CLDN7**	1.5482503	1.8133206	1.81529316	1.93106687	1.776982733
**LSM4**	1.576591	2.2202434	2.14587955	1.61180081	1.88862869
**PTTG1**	1.5851681	1.7241335	2.53092828	2.81618777	2.164104413
**ASS1**	1.5863852	1.7809022	2.26650699	2.5761963	2.052497673
**UCP2**	1.5886987	1.9341559	2.79684603	2.45453011	2.193557685
**HIST2H2AA4**	1.6037393	3.8132994	1.57163767	1.92279805	2.227868605
**GALNT6**	1.6283555	1.5639289	1.89393225	2.2184833	1.826174988
**SPON1**	1.6343724	1.7557265	2.61555009	5.11917689	2.78120647
**CDKN2A**	1.6582585	1.8668662	1.56889283	4.87824132	2.493064713
**TNNT1**	1.7120016	1.7248377	2.59199335	2.90556476	2.233599353
**IFI27**	1.7304991	2.0389137	2.42325374	1.53066183	1.930832093
**CXCR4**	1.7323896	1.5529248	3.09185858	2.61428996	2.247865735
**CCNE1**	1.8135302	1.727517	1.8645167	3.70945345	2.278754338
**GINS1**	1.8504189	1.5347887	3.28216941	2.22135516	2.222183043
**SLC39A4**	1.8591187	2.042175	2.22964269	1.84784703	1.994695855
**ADGRG1**	1.8736437	2.2297834	2.77079181	3.66756572	2.635446158
**VTCN1**	1.8750525	1.5188603	2.55932736	1.91721732	1.96761437
**RACGAP1**	1.889408	1.6151755	2.61099198	2.38066859	2.124061018
**HIST1H1C**	2.0136981	2.0258376	2.3777018	2.18210599	2.149835873
**UBE2C**	2.0259988	2.1169556	2.27206271	2.53923324	2.238562588
**PFKP**	2.0560559	1.5970903	2.92923891	1.69037224	2.068189338
**SORT1**	2.0706019	1.7272631	2.19047725	3.18517285	2.293378775
**MTHFD2**	2.0761211	1.855699	3.43802822	2.96200561	2.582963483
**SCGB1D2**	2.1014059	1.9011465	1.87980413	2.36715655	2.06237827
**S100A13**	2.1201662	2.6353998	2.12671922	2.11842323	2.250177113
**SLC52A2**	2.1858062	2.5925358	1.58759595	2.79062935	2.289141825
**CLDN4**	2.1874141	2.1017913	2.87235227	2.05624714	2.304451203
**IDH2**	2.1961069	2.0583116	3.24694425	1.51743071	2.254698365
**TRIP13**	2.2153919	1.7864702	3.80169245	2.77658013	2.64503367
**SST**	2.2553965	1.6702669	2.7368555	3.61622586	2.56968619
**PAX8**	2.2673437	1.9338173	1.87104122	2.72704711	2.199812333
**DEFB1**	2.295469	2.541294	3.17608453	3.50199123	2.87870969
**CKS2**	2.3528225	2.362744	3.25360268	2.86869791	2.709466773
**KIAA0101**	2.3755858	1.5843077	3.3531618	3.0426095	2.5889162
**MUC1**	2.4182557	2.9103658	3.71077769	5.00959119	3.512247595
**KLK8**	2.436599	2.031964	2.84211498	3.31858194	2.65731498
**TOP2A**	2.4917396	1.7114638	3.90640641	3.15280703	2.81560421
**DDX17**	2.5173586	-2.8884915	-3.00669809	-1.85887144	-1.309175608
**CLDN10**	2.5491458	1.570669	2.32710887	1.61025382	2.014294373
**LCN2**	2.5508985	2.5979465	2.63279129	2.60555165	2.596796985
**SOX9**	2.5661536	2.2188302	3.06109474	2.47479424	2.580218195
**GLDC**	2.5982669	1.8639102	2.25822023	4.01894894	2.684836568
**CRABP2**	2.6030514	3.3120457	3.75504242	2.09301216	2.94078792
**EPCAM**	2.6076583	1.9374149	3.91796973	2.90958647	2.84315735

**CDC20**	2.629348	2.0688945	2.67333309	2.44011922	2.452923703
**RGS1**	2.6920854	1.6183767	2.94789518	3.20990236	2.61706491
**ELF3**	2.7166184	2.6216772	3.4397343	3.20214881	2.995044678
**S100A2**	2.7391937	2.4057574	2.52216025	3.78688002	2.863497843
**C1orf106**	2.7471391	2.0594274	3.22704702	3.2938179	2.831857855
**INHBB**	2.7672152	1.5396394	1.72120248	2.28741927	2.078869088
**CDK1**	2.7712418	1.558293	4.24011987	2.55175108	2.780351438
**PRAME**	2.8031508	1.7097868	2.84741732	4.11650071	2.869213908
**KLK6**	2.8093839	2.5609309	3.9628796	2.39721001	2.932601103
**TTK**	2.8357612	1.727283	3.5955271	3.14724284	2.826453535
**LYPD1**	3.0058514	2.0988132	3.78894049	3.14999875	3.01090096
**MAL**	3.0796118	1.9481591	3.39921118	4.60962458	3.259151665
**FXYD3**	3.0898327	2.127299	1.59995322	1.71196547	2.132262598
**FOXM1**	3.2642259	1.6858633	2.47867993	2.86265696	2.572856523
**WFDC2**	3.2909179	3.6042786	3.74623999	3.17675115	3.45454691
**NEK2**	3.3192172	1.8704568	4.11000516	3.12679342	3.106618145
**FOLR1**	3.553973	3.4741996	4.26986154	3.82733819	3.781343083
**CLDN3**	3.6653647	2.7608509	4.34375773	2.80673374	3.394176768
**MMP7**	3.8098733	2.1593555	4.5903028	3.09040775	3.412484838
**SOX17**	3.8447394	3.0739197	4.66537404	5.25235079	4.209095983
**CP**	3.8582713	2.3290776	5.50114568	5.53471225	4.305801708
**LOC101929219**	3.9232044	3.8807065	4.5350819	3.72002537	4.014754543
**SCGB2A1**	4.6035392	3.5918179	4.57503223	2.97618963	3.93664474
**HMGA1**	4.7378028	2.4695224	2.38238349	3.32207276	3.227945363
**MECOM**	4.9760337	2.8703197	6.5092766	4.93259787	4.822056968
**CD24**	5.8941727	4.3455616	5.94921647	4.06978934	5.064685028

Common downregulated DEGs: adjusted p-value < 0.05 and log fold change (FC) > 1.5

**Table S2.**	**The common downregulated gene signature in ovarian cancer and gene FC values.**				
	GSE18520	GSE26712	GSE54388	GSE14407	Average FC
ADH1B	-5.7689581	-3.5281206	-5.3828649	-5.80156146	-5.120376265
BCHE	-5.1106175	-4.4300515	-5.57631282	-5.51658163	-5.158390863
ABCA8	-5.0699307	-4.167755	-6.96328264	-6.42840621	-5.657343638
MEOX2	-5.0493452	-1.6499411	-2.95841772	-4.36060991	-3.504578483
NPY1R	-4.8848776	-3.6719391	-5.26073126	-3.49384941	-4.327849343
AOX1	-4.7958022	-4.225605	-2.24233008	-4.90599851	-4.042433948
BNC1	-4.7909113	-3.7299279	-6.09482574	-7.10951987	-5.431296203
REEP1	-4.6773319	-3.9476068	-4.96730013	-5.88106708	-4.868326478
HBB	-4.6708376	-4.098042	-6.16593334	-7.13317592	-5.516997215
OGN	-4.6253585	-2.3691737	-5.69189394	-6.56325754	-4.81242092
LOC101928635	-4.5466848	-4.36511	-4.92487227	-8.00200421	-5.45966782
TMEM255A	-4.5009466	-3.3276946	-3.91908094	-4.24674475	-3.998616723
EFEMP1	-4.4229358	-3.8974165	-4.60273709	-4.96720795	-4.472574335
TCF21	-4.3466745	-2.3007954	-3.74501827	-3.94867812	-3.585291573
DIRAS3	-4.0892636	-2.7786717	-4.73425672	-2.28874098	-3.47273325
SLC4A4	-4.0699488	-3.6705102	-4.73585685	-4.59918334	-4.268874798

MNDA	-4.0376835	-4.4397401	-4.60843391	-3.54734023	-4.158299435
SPOCK1	-4.0214031	-3.2039716	-4.2052485	-4.82941626	-4.065009865
RNF128	-4.0039641	-1.7183562	-3.95217824	-3.44529379	-3.279948083
MAF	-3.9921298	-3.1844745	-4.16248842	-2.0688948	-3.35199688
PROCR	-3.988991	-3.4770387	-3.79514362	-2.9635651	-3.556184605
MS4A4A	-3.9786197	-1.9668183	-1.80516399	-1.50624573	-2.31421193
PKD2	-3.9168222	-2.204135	-2.36851297	-2.15975628	-2.662306613
LOC101930363	-3.9135661	-4.6619823	-4.77908792	-4.26894378	-4.405895025
HBA2	-4.1059935	-3.5716722	-3.54885038	-5.62094873	-4.211866203
PRG4	-3.8413263	-2.9544698	-5.12172703	-5.48447143	-4.35049864
TCEAL2	-3.8054457	-4.0664253	-5.52578156	-4.51920071	-4.479213318
HAS1	-3.7968813	-2.0272248	-3.1309992	-1.91658994	-2.71792381
CHRDL1	-3.7841946	-3.614563	-4.00264759	-5.4232821	-4.206171823
PCOLCE2	-3.7725582	-2.225823	-2.66151977	-2.74261986	-2.850630208
GPM6A	-3.6705417	-3.8586356	-3.88307951	-4.83692642	-4.062295808
METTL7A	-3.6571823	-2.5568076	-1.62707612	-3.18563414	-2.75667504
WNT2B	-3.6521153	-2.667646	-5.89187918	-5.93037063	-4.535502778
CHGB	-3.6120564	-2.0739057	-3.89881556	-3.17849533	-3.190818248
HBG2	-3.6047161	-2.4523458	-1.6606096	-4.12785608	-2.961381895
PTGER3	-3.5877389	-1.9384261	-3.36163675	-2.7321125	-2.904978563
LGALS8	-3.5532435	-3.2684735	-1.82345085	-2.80145869	-2.861656635
GHR	-3.5307382	-2.5214713	-3.88025766	-3.35998722	-3.323113595
NKX3-1	-3.4605137	-2.5772292	-3.29539649	-3.87431786	-3.301864313
PTPRZ1	-3.4468394	-1.5675705	-2.42560509	-3.5199432	-2.739989548
CSGALNACT1	-3.4272192	-2.3194621	-3.40385698	-4.01198779	-3.290631518
LOC100506718	-3.3823064	-1.9763368	-3.14091209	-3.83600071	-3.083889
PTPN13	-3.3714943	-1.5636582	-1.79438582	-1.65181362	-2.095337985
ANXA8L1	-3.3327289	-3.4003146	-3.92543041	-4.96028156	-3.904688868
LHX2	-3.3193626	-3.7502874	-5.36122155	-6.39499766	-4.706467303
PDGFD	-3.2730175	-2.9978876	-3.92962725	-4.58076949	-3.69532546
TSPAN8	-3.2563743	-2.2687633	-4.00737502	-2.86283953	-3.098838038
DFNA5	-3.2550773	-3.3406929	-3.76671645	-3.40760806	-3.442523678
CLEC4M	-3.7083967	-2.4698838	-4.17337639	-3.43725993	-3.447229205
LGALS2	-3.223351	-2.1992862	-2.95543194	-4.80514857	-3.295804428
SFRP1	-3.2206553	-3.1809029	-3.63235441	-5.88642216	-3.980083693
DPYD	-3.2144845	-3.7205607	-3.42276345	-3.63476051	-3.49814229
PMP22	-3.1827726	-1.7615297	-2.42194004	-3.4835449	-2.71244681
PEG3	-3.1772001	-3.1552952	-3.40330699	-3.57268559	-3.32712197
TFPI	-3.1569535	-1.8621495	-2.57695366	-2.89318908	-2.622311435
DCN	-3.1257613	-2.1511781	-2.2531398	-4.90639447	-3.109118418
HSD17B6	-3.0608702	-1.5735179	-2.0674626	-2.03414089	-2.183997898
GFPT2	-3.0527749	-2.2455099	-2.83074799	-4.12102703	-3.062514955
KDR	-3.0295542	-3.1167568	-4.0369898	-1.85058766	-3.008472115
DSC3	-3.0171774	-2.2612733	-1.83174524	-4.61968473	-2.932470168
NAP1L3	-3.0133026	-3.28136	-3.70219777	-2.98685917	-3.245929885
ZFPM2	-3.0013691	-2.7590526	-2.46205136	-2.94956776	-2.793010205
MARCO	-2.9981814	-2.7049442	-2.31924981	-2.95761146	-2.744996718
ALDH1A1	-2.9272267	-2.7791164	-4.07699112	-2.48179951	-3.066283433

PLCE1	-2.9233592	-2.227662	-2.55483729	-2.90147016	-2.651832163
CMAHP	-2.9222435	-1.6204149	-2.91980654	-2.45316592	-2.478907715
PDE8B	-2.8514563	-2.3989605	-4.12332578	-3.81593542	-3.2974195
LOC101926921	-2.873467	-2.8992213	-3.80844961	-3.10960613	-3.17268601
TFPI2	-2.8560032	-3.0690163	-1.95208238	-4.39322888	-3.06758269
CLDN15	-2.8076187	-2.2505559	-2.8454579	-2.62286443	-2.631624233
AKT3	-2.8058384	-1.7705263	-1.96869667	-2.44181334	-2.246718678
FGF13	-2.8025683	-2.7593924	-3.06727065	-3.68852571	-3.079439265
ECM2	-2.7943268	-2.5634693	-3.57275345	-2.67636103	-2.901727645
LAMA4	-2.7828376	-2.7142826	-3.48599898	-2.35888682	-2.8355015
NELL2	-2.7818707	-3.6844362	-3.50380464	-4.86403691	-3.708537113
CAV1	-2.7796538	-2.8699554	-2.84038132	-3.20605073	-2.924010313
RARRES1	-2.7719759	-2.0155122	-3.6526643	-5.04666439	-3.371704198
SNCAIP	-2.7626101	-2.6950327	-3.0757177	-3.0251148	-2.889618825
NBR1	-2.7500152	-1.9142875	-1.70025683	-2.49524544	-2.214951243
AQP9	-2.7246993	-2.729174	-2.67301937	-4.29389584	-3.105197128
CALB2	-2.685319	-3.8984394	-3.9595774	-3.57006268	-3.52834962
GPRASP1	-2.6799383	-2.3153749	-3.94997008	-2.71393586	-2.914804785
FLRT3	-2.6744304	-2.2627885	-2.90402592	-2.8416682	-2.670728255
THBD	-2.6519397	-1.6788064	-3.7289014	-2.48790765	-2.636888788
EZR	-2.5838277	-2.0105635	-2.47846167	-1.97725397	-2.26252671
ADAMTS3	-2.5670983	-2.1379239	-2.23863392	-2.24608338	-2.297434875
PITPNC1	-2.5650602	-1.6005441	-2.16089765	-3.49057977	-2.45427043
FRY	-2.5631801	-3.1025089	-3.3120786	-3.2486448	-3.0566031
CFH	-2.5430897	-2.265457	-3.51303448	-3.57782058	-2.97485044
CYP39A1	-2.5317523	-1.9543535	-3.04317984	-2.66135773	-2.547660843
FGF9	-2.528293	-2.4197006	-2.89533577	-4.88263448	-3.181490963
PAPSS2	-2.522264	-1.6802825	-3.13367323	-2.99172212	-2.581985463
SEMA5A	-2.5121815	-2.2633663	-2.24426599	-3.16810505	-2.54697971
MTUS1	-2.5005844	-1.8706449	-1.80705218	-3.18640054	-2.341170505
DST	-2.4933966	-1.5901907	-2.4785004	-2.30328867	-2.216344093
PTGDR	-2.484622	-1.7212115	-3.14642929	-2.99651533	-2.58719453
IL18	-2.4784675	-1.9172985	-2.88760401	-2.9316339	-2.553750978
MDFIC	-2.4721966	-1.7869615	-2.02952015	-2.22918803	-2.12946657
RUNX1T1	-2.454723	-1.694563	-2.90837196	-2.70260195	-2.440064978
LY75-CD302	-2.447611	-1.7113087	-2.49229138	-2.03982106	-2.172758035
ME1	-2.4400698	-2.2779682	-2.61370455	-1.99017908	-2.330480408
CFI	-2.4308269	-2.5887773	-2.8740432	-2.24252105	-2.534042113
CFHR1	-2.4216438	-2.3519407	-2.98851954	-3.16226855	-2.731093148
OLFML1	-2.411794	-2.4423333	-3.44400126	-2.86059574	-2.789681075
RGS4	-2.3894472	-2.8681307	-3.45284006	-4.34521394	-3.263907975
SNCA	-2.3562038	-1.8337609	-2.62146302	-3.75422659	-2.641413578
BAMBI	-2.3550641	-2.0492038	-2.7974974	-2.76162914	-2.49084861
RNASE4	-2.3550401	-2.9258476	-3.12436782	-4.35890658	-3.191040525
RTN1	-2.3487017	-2.9487552	-3.68654278	-3.28499012	-3.06724745
PCDH9	-2.33076	-2.2026314	-3.32238812	-3.55331261	-2.852273033
SOBP	-2.3251738	-2.2668254	-2.7876694	-1.93424398	-2.328478145
FAM134B	-2.3181214	-1.6977143	-2.00125745	-2.25912274	-2.069053973

NBEA	-2.3137808	-1.7971695	-2.13429397	-3.21837491	-2.365904795
PROS1	-2.3024784	-3.3456003	-2.6693135	-2.80470146	-2.780523415
TLE4	-2.3003883	-2.3826089	-2.37828986	-3.14254374	-2.5509577
PDZD2	-2.272068	-2.0424062	-2.30637125	-1.87307652	-2.123480493
DSE	-2.2704122	-3.0794871	-2.56603839	-2.72775832	-2.660924003
MAP3K8	-2.2229769	-2.1568196	-2.37106844	-1.93772264	-2.172146895
WNT5A	-2.213879	-2.7865746	-2.64793195	-2.3590869	-2.501868113
ATP10D	-2.1956486	-2.2716214	-2.3393752	-1.97011496	-2.19419004
NR2F1	-2.1910427	-2.6901325	-3.26385347	-1.61296625	-2.43949873
TRPC1	-2.1875945	-2.4760254	-2.273646	-3.04079459	-2.494515123
GAS1	-2.1755669	-2.7071004	-2.31248411	-2.70266651	-2.47445448
GATA6	-2.1750218	-2.8642411	-3.23843398	-3.5061217	-2.945954645
PRKAR2B	-2.1636952	-2.1382866	-2.67385168	-3.43492415	-2.602689408
NDN	-2.1599629	-1.7096975	-2.25406741	-3.58742816	-2.427788993
KLF4	-2.1598107	-2.2234175	-2.29688231	-2.17010795	-2.212554615
SVEP1	-2.1557503	-1.9123177	-2.67048001	-2.99566033	-2.433552085
NR3C1	-2.1550624	-1.5743244	-2.05492202	-1.76058681	-1.886223908
BDH2	-2.1447209	-1.5553481	-1.9247253	-2.42635001	-2.012786078
STK26	-2.130086	-3.9748483	-2.15872108	-2.74393067	-2.751896513
NEFH	-2.1128637	-1.659517	-3.42381321	-3.62072264	-2.704229138
ARHGAP44	-2.1064333	-2.2321754	-1.57111022	-2.88151447	-2.197808348
MEIS2	-2.0901766	-3.0521864	-2.9243863	-2.63055437	-2.674325918
NT5E	-2.0898327	-1.7522724	-2.24618672	-2.41273501	-2.125256708
DMD	-2.0594746	-2.6265138	-2.87924471	-2.52626101	-2.52287353
HSD17B2	-2.0541438	-1.695097	-3.22911842	-4.38080171	-2.839790233
NRXN3	-2.0483808	-1.8797824	-1.88267886	-2.21371837	-2.006140108
CELF2	-2.0265593	-1.8941702	-1.78085663	-2.76314027	-2.1161816
S100A8	-2.0139143	-1.9505706	-2.49715726	-3.47703517	-2.484669333
NLGN4X	-2.0123171	-1.5507078	-3.01931956	-3.27891271	-2.465314293
FAM65B	-1.9934088	-1.741029	-2.03725701	-2.89857133	-2.167566535
PDPN	-1.9811827	-2.1431935	-2.40020889	-2.07143389	-2.149004745
PTGIS	-1.9727192	-2.6032643	-4.13608951	-3.6092443	-3.080329328
PDGFRA	-1.946443	-1.877806	-3.12885339	-2.66716368	-2.405066518
SLC16A1	-1.9395554	-1.7375157	-1.64052501	-2.27683985	-1.89860899
RUFY3	-1.9307561	-1.9798619	-2.37664168	-1.63699689	-1.981064143
TSPYL1	-1.9214133	-2.158204	-1.60361875	-1.86267603	-1.88647802
MAOA	-1.9143216	-1.5146756	-2.51169302	-2.77731105	-2.179500318
MEF2C	-1.9134633	-2.0122845	-2.17613266	-2.15041217	-2.063073158
GREB1	-1.9057264	-2.6941656	-2.18580463	-1.90860608	-2.173575678
TPBG	-1.8871181	-1.9648569	-1.85990795	-3.06432424	-2.194051798
SLC39A8	-1.8733146	-3.6203723	-1.6618424	-2.96287738	-2.52960167
HPR	-1.8485719	-2.1899093	-1.6827256	-2.57977173	-2.075244633
WASF3	-1.8444519	-1.574059	-1.94139144	-1.88145675	-1.810339773
CAV2	-1.8361671	-2.213583	-2.67598618	-3.95632092	-2.6705143
C21orf62	-1.8321227	-2.1125561	-2.37297825	-4.4410803	-2.689684338
SCG5	-1.8269467	-2.758728	-2.17113737	-2.77410335	-2.382728855
PLSCR4	-1.8267321	-3.3925309	-2.22180416	-3.39076702	-2.707958545
ARHGEF10	-1.8115547	-1.5518986	-1.62415165	-1.88365898	-1.717815983

HSPA2	-1.8084577	-1.7776543	-1.88411749	-1.87549267	-1.83643054
LEPROT	-1.7750975	-1.5226618	-2.36066818	-1.61660988	-1.81875934
HNMT	-1.7717997	-1.6061011	-2.2034673	1.67026711	-0.977775248
GNG11	-1.7513487	-2.1442506	-2.09791818	-2.41020412	-2.1009304
ABHD11	-1.7447011	1.9988828	2.40174553	1.55047269	1.05159998
TACC1	-1.7071604	-2.5505833	-1.92230942	-3.79557497	-2.493907023
ANOS1	-1.6835518	-2.1989315	-1.81321328	-2.05974327	-1.938859963
LAMB1	-1.6809285	-2.2348326	-1.7171636	-1.67354923	-1.826618483
TNXB	-1.666131	-1.5553633	-2.49408056	-2.07824889	-1.948455938
ARMCX1	-1.6660126	-3.4513774	-1.93375991	-2.206937	-2.314521728
CPE	-1.6601362	-2.5219973	-2.62433233	-2.87113103	-2.419399215
BCL2	-1.6570289	-1.9702963	-1.98492107	-3.34831023	-2.240139125
TXNIP	-1.6547952	-1.8583254	-2.29506141	-4.08618642	-2.473592108
FHL1	-1.6545908	-1.8468289	-1.91331545	-2.34821719	-1.940738085
QKI	-1.6518229	-2.573701	-1.57605812	-1.59709774	-1.84966994
TMX4	-1.6488609	-2.7922047	-1.66726559	-2.07412248	-2.045613418
PHLPP2	-1.6465789	-1.9356768	-1.93691178	-2.73297622	-2.063035925
SLC31A2	-1.6416207	-1.8139472	-1.657694	-2.66315406	-1.94410399
SMARCA2	-1.6357337	-2.0729893	-1.5694402	-3.59659497	-2.218689543
HEG1	-1.6332696	-3.147903	-1.63038451	-1.68617762	-2.024433683
N4BP2L2	-1.6325208	-2.8265002	-2.23064322	-3.60373773	-2.573350488
RECK	-1.6248593	-2.3871977	-2.97165138	-1.57672744	-2.140108955
TRA2A	-1.6020422	-1.7861149	-2.37089855	2.28990871	-0.867286735
MAN1A1	-1.6004038	-1.9273587	-2.20307298	-1.6420505	-1.843221495
AMIGO2	-1.5680417	-2.7964589	-1.7096788	-2.75485993	-2.207259833
SEMA3C	-1.5606831	-2.5230971	-2.36095918	-3.85921819	-2.575989393
PDLIM5	-1.5490674	-1.6840456	-1.56661365	-2.00239726	-1.700530978
NDNF	-1.5400828	-1.7955413	-2.89853529	-3.99193984	-2.556524808
LOC728392	-1.5368559	-1.7369442	-1.73969752	-2.03452578	-1.76200585
SERPINB9	-1.535668	-2.134485	-1.50665728	-1.86124808	-1.75951459
GPR137B	-1.514345	-2.1528251	-1.55942001	-1.69627783	-1.730716985

Common downregulated DEGs: adjusted p-value < 0.05 and log fold change (FC) > 1.5

Table S3: Enriched KEGG Pathways

#term ID	term description	strength	FDR
hsa04110	Cell cycle	1.12	0.00063
hsa04670	Leukocyte transendothelial migration	1.08	0.0028
hsa04514	Cell adhesion molecules (CAMs)	0.99	0.005
hsa04114	Oocyte meiosis	0.97	0.0201
hsa05160	Hepatitis C	0.92	0.0248
hsa04115	p53 signaling pathway	1.08	0.0305
hsa01230	Biosynthesis of amino acids	1.05	0.0306
hsa04218	Cellular senescence	0.84	0.0306
hsa04530	Tight junction	0.81	0.0306
hsa05200	Pathways in cancer	0.57	0.0306
hsa05203	Viral carcinogenesis	0.77	0.0364
hsa05222	Small cell lung cancer	0.95	0.0364

Table S4: Enriched Gene Ontology (Biological Process)			
#term ID	term description	strength	FDR
GO:2000241	regulation of reproductive process	1.02	0.0016
GO:0007292	female gamete generation	0.94	0.0176
GO:0001824	blastocyst development	1.25	0.0036
GO:0007143	female meiotic nuclear division	1.46	0.0056
GO:0032268	regulation of cellular protein metabolic process	0.36	0.0052
GO:0042493	response to drug	0.52	0.0094
GO:0002376	immune system process	0.29	0.0413
GO:0044703	multi-organism reproductive process	0.62	0.00055
GO:0019953	sexual reproduction	0.59	0.0039
GO:0030858	positive regulation of epithelial cell differentiation	1.14	0.0188
GO:0034501	protein localization to kinetochore	1.59	0.0188
GO:0071599	otic vesicle development	1.59	0.0188
GO:0007568	aging	0.81	0.0082
GO:0046655	folic acid metabolic process	1.48	0.0261
GO:0060429	epithelium development	0.41	0.0428
GO:0071902	positive regulation of protein serine/threonine kinase activity	0.68	0.0201
GO:0009790	embryo development	0.44	0.0434
GO:0051983	regulation of chromosome segregation	1.23	0.00026
GO:0051988	regulation of attachment of spindle microtubules to kinetochore	1.66	0.0164

**Table S5: ADMET, safety/toxicity and drug likeness profile of NSC777201**		
**Properties**	**NSC777201**	**Reference value**
Formula	C21H22ClN3O2S	-
Molecular weight	415.94 g/mol	150-500 g/mol
Num. rotable bonds	7	0-9
Num. H-bond acceptors	4	0-10
Num. H-bond donors	3	0-5
Molar Refractivity	116.96	-
TPSA	99.02 A2	20 to 130 A2
Fraction Csp3	0.33	0.25<1
Log P o/w (XLOGP3)	3.13	minus 0.7 to 5
Consensus Log P o/w	3.3	-
Log S (ESOL)	-4.25	0-6
Lipinski, Ghose, Veber and Egan's rule	Yes, 0 violation	-
Bioavailability Score	0.55	>0.1 (10%)
Synthetic accessibility	4.57	1 (very easy) to 10 (very difficult)
**Acute Toxicity**		
LD50 for Intraperitoneal (mg/kg)	547,100 (OECD: 5)	
LD_{50} for Intravenous (mg/kg)	76,580 (OECD: 4)	
LD_{50} for Oral (mg/kg)	958,100 (OECD: 4)	

LD_{50} for Subcutaneous (mg/kg)	492,400 (OECD: 4)
**Environmental Toxicity**	
Bioaccumulation factor Log10 (BCF)	1,230
Daphnia magna LC_{50} Log10 (mol/L)	7,028
Fathead Minnow LC_{50}Log10 (mmol/L)	-3,765
Tetrahymena pyriformis IGC_{50}Log10 (mol/L)	1,732
